# Editorial: The morphology and physiology of insect chemosensory systems – Its origin and evolution

**DOI:** 10.3389/fnana.2022.1024927

**Published:** 2022-09-29

**Authors:** Rui Tang, Xin-Cheng Zhao

**Affiliations:** ^1^Guangdong Key Laboratory of Animal Conservation and Resource Utilization, Guangdong Public Laboratory of Wild Animal Conservation and Utilization, Institute of Zoology, Guangdong Academy of Sciences, Guangzhou, China; ^2^Henan International Laboratory for Green Pest Control/College of Plant Protection, Henan Agricultural University, Zhengzhou, China

**Keywords:** insect, chemosensory system, morphology, physiology, neural pathway, omics

Insects rely heavily on the ancient chemosensory modality to colonize in the environment. To date, the huge diversity of insect species and their special adaptations to distinct ecological niches are mostly driven by key environmental cues, species-specific neural pathways, and functional repertoires of several biological macromolecule classes. These essential elements contain an indispensable clue from which modern phenotype-to-molecular study scenarios were established. The chemosensory circuits and physiological traits in insect species can reflect protein-ligand interactions at the molecular level and *vice versa*. As such, the omic-based reverse chemical ecology has been swiftly improved along with the classic chemical ecology. Information on either side can help us gain knowledge of the other side, and provide information to help solve the riddle of how certain chemosensory traits emerged and evolved in insects. This Research Topic aims to seek morphological, physiological, and molecular features in insect chemosensory system toward allelochemicals and provide the linkage between architectures and innervated/environmental causes which forged the insect populations over time.

## Inter- and intra-species communication

Pre-mating sex communication in Lepidoptera is one of the most representative modules to understand chemical-driven behavioral decisions in insects. In the sex pheromone detection processes, sex pheromone molecule (SPM), pheromone receptor (PR), co-receptor (ORco), pheromone binding protein (PBP), sensory neuron membrane protein (SNMP), and pheromone degradation enzyme (PDE) play individual and cooperative roles. The review from Yang et al. summarized SPR/ORco from 10 families of Lepidopteran insects regarding the receptor-ligand clustering, protein topologies, and updated pheromone signaling pathways. Future interests of the area are expected to explore the cryo-EM structure of SPR and the SPR-ligand docking pattern from a biophysical perspective.

The function of peripheral neurons in recognizing sex pheromones provides fundamental knowledge for us to link species ecology with biological molecules and innervated circuits. The work of Wang et al. filled this gap in the oriental armyworm *Mythimna separata*. The trichoid sensilla (TS) from both sexes of adults were classified into four types after carrying out single sensillum recording (SSR) tests. The neurons within the TS were further characterized, and corresponding PRs were speculated according to later functional deorphanizations of receptors.

The odorant coding to aromatics in insect pollinators is important in understanding insect-plant interactions. Dong et al. studied the syrphid fly *Eupeodes corollae* in terms of antennal sensillar clustering, ultrastructural characterization, and SSR firing patterns, by providing results from SEM, TEM, and SSR, respectively. Sensillar types and structures of the species were described in detail, and a sensilla basiconica (SB) answering to methyl eugenol was identified.

The host acceptances of insects can be determined largely by detecting plant metabolites with taste. Sun et al. showed that larvae of the generalist *Helicoverpa armigera* and the specialist *H. assulta* had distinct herbivore inducing and responding patterns toward host molecules. Components including saccharides, amino acids, and other secondary metabolites elicited varied feeding responses between the two species. The firing patterns of gustatory receptor neurons (GRNs) in the maxillary styloconic sensilla were found to be consistent with the differences in feeding preferences between the two species.

## Insect chemosensory genes in the era of omics

Development and host ranges influence gene expressions in insects. The work presented by Tian et al. utilized weighted gene co-expression network analysis (WGCNA) to construct gene co-expression networks in the whitefly *Bemisia tabaci*. Co-expression modules related to host plant selection were identified by cross checking with transcriptomic data from various tissues.

The mature RNA-seq technology is by far the most convenient way to tackle spatial-temporal functional annotations of chemosensory genes in insects. The work from Liu et al. analyzed tissue- and gender-wise transcriptome of *Chilo sacchariphagus* and identified a panel of comprehensive chemoreception-related family genes. Annotations were further evaluated by phylogenetic analysis and real-time expression profiling.

It is essential to link peripheral architectures with associated chemosensory genes. The work on the orange spiny whitefly *Aleurocanthus spiniferus* provided by Gao et al. unveiled antennal sensillar types and chemosensory gene annotations of the species. The expression profiling of selected chemosensory genes at different developmental stages was further examined and mapped to transcriptomes.

Aldehyde oxidases (AOXs) are common detoxifying enzymes in several organisms. In insects, AOXs act in xenobiotic metabolism and as odorant-degrading enzymes (ODEs). In the work of Godoy et al., novel *AOX* families were reported by checking 18 genomes from moths and butterflies. Odorant-degrading functions of the two clades were estimated through phylogenetic tests, involving both plant volatiles and sex pheromones, respectively.

In insects, odorant binding proteins (OBPs) form a vital chemosensory family which is involved in transporting hydrophobic odor molecules from the external environment to receptor neurons. By providing a comparative genomic analysis using the codling moth *Cydia pomonella*, Huang et al. described evolution traits of *OBPs* in the species. Possible distant ancestral OBPs were found lost, and the expansion of OBPs was speculated to have resulted from tandem duplications.

Ligand binding properties are major concerns in exploring insect OBPs. Jiang et al. characterized this family within *Aphidius gifuensis*, which was the most common endoparasitoids of the field aphids, by providing evidence from transcriptomes, expression profiling, gene cloning, and competitive ligand binding assays. It showed that OBPs abundantly expressed in the legs of this species were responsible for binding an analog of (E)-β-farnesene, which has a known function in aphid ecology.

Insect-plant interactions are also initiated from protein-ligand binding of OBPs in herbivores. Hong et al. identified an antennal-specific OBP within the jujube bud weevil *Pachyrhinus yasumatsui via* transcriptomic analysis, and further evaluated this protein in a binding test. A broad ligand recognition pattern was found with the tested OBP, and essential amino acid residues for binding were estimated *via* modeling and docking simulations.

## Expanding methodologies and aspects

Electrophysiological tests can never be too precise. Li et al. reported on a new electroantennogram (EAG) recording technique for evaluation of electrophysiological responses of antennal lamellae of *Pseudosymmachia flavescens* to sex pheromones and host-plant-related compounds. EAG responses were recorded simultaneously from each lamella and the closed antennal club. This has provided a method to separate EAG channels of lamellae in the scarab beetles or other possible insects with unconventional antennal morphology.

Land to freshwater transitions occurred in many lineages within the insect tree of life. Whether chemosensory gene repertoires of aquatic insects remained essentially unchanged or underwent more or less drastic modifications to cope with physico-chemical constraints associated with life underwater remains virtually unknown. Montagné et al. provided the transcriptome of chemosensory organs of the diving beetle *Rhantus suturalis* and described OBP and OR expansions specific to diving beetles. These duplicated genes tend to be expressed in palps rather than in antennae, suggesting a possible adaptation with respect to the land-to-water transition.

Chemosensory-related genes are expressed in various tissues, including non-sensory organs, and they play diverse roles. Insect OBPs and CSPs have been detected in various tissues as well as olfactory organs. They are involved in carrying semiochemicals that have various roles, such as in reproduction, regeneration, development, nutrition, anti-inflammatory action, and vision. The work by Chen et al. showed that OBPs and CSPs existed in the brains of oriental armyworm adults, by providing transcriptomic analysis and sex-biased expression profiling. This may help us understand novel functions and ligand targets of insect chemosensory proteins.

Neuroanatomical studies are regularly done in adults of agricultural pests, but brain structures from larvae are rarely reported. Zhang et al. targeted the important fall armyworm species and traced the serotonergic and serotonergic neural networks within the larval brain and gnathal ganglion. Morphological mapping and segmentation were conducted with the above neuropils of the species. Wiring of serotonergic neurons were described in detail.

## Conclusion

To conclude, the current hot spots in insect chemosensory research include pheromone-based behavioral driving and multi-trophic interaction mechanisms. Next-generation omics and iterative analytic tools have granted us vast data to explore and a unique chance to trace evolutionary milestones among species. We have harvested novel results, views, and prospects within this topic that they will inspire further studies on precise neural pathways, functional deorphanizations, and adaptation history of chemoreception in insects.

## Author contributions

RT and X-CZ drafted and finalized this document.

## Funding

This report was supported by Guangzhou Science and Technology Project (No. 202201010039) and GDAS Special Project of Science and Technology Development (2022GDASZH-2022010106).

## Conflict of interest

The authors declare that the research was conducted in the absence of any commercial or financial relationships that could be construed as a potential conflict of interest.

## Publisher's note

All claims expressed in this article are solely those of the authors and do not necessarily represent those of their affiliated organizations, or those of the publisher, the editors and the reviewers. Any product that may be evaluated in this article, or claim that may be made by its manufacturer, is not guaranteed or endorsed by the publisher.

